# Lithium-Induced Bradycardia and Cardiomyopathy in a Patient With Bipolar Disorder and Paranoid Schizophrenia

**DOI:** 10.7759/cureus.40949

**Published:** 2023-06-25

**Authors:** Mahnoor Khalid, Wasiq Sheikh, Mahnoor Sherazi, Tasnim F Imran

**Affiliations:** 1 Internal Medicine, Foundation University Medical College, Islamabad, PAK; 2 Internal Medicine, Cardiology, Lifespan Cardiovascular Institute, Providence, USA; 3 Cardiology, Lifespan Cardiovascular Institute, Providence, USA; 4 Cardiology, Warren Alpert Medical School of Brown University, Providence, USA; 5 Internal Medicine, State University of New York (SUNY) Upstate Medical University, Syracuse, USA; 6 Cardiology, Providence VA Medical Center, Warren Alpert Medical School of Brown University, Providence, USA

**Keywords:** chronic lithium therapy, lithium poisoning, lithium-induced bradycardia, drug-induced cardiomyopathy, lithium induced cardiotoxicity

## Abstract

Lithium is primarily known to cause neurological and gastrointestinal side effects, however, cardiac effects have been rarely reported. We present a unique case of lithium cardiotoxicity causing bradyarrhythmia and cardiomyopathy. A 68-year-old man with a history of paranoid schizophrenia and bipolar disorder presented with altered mental status. On examination, the patient was lethargic, afebrile, with dry oral mucosa, and a regular pulse of 42 bpm. Labs revealed acute kidney injury and elevated lithium levels. Electrocardiogram (ECG) revealed a junctional escape rhythm with a right bundle morphology. Lithium toxicity was strongly suspected in the setting of raised serum lithium levels, decreased oral intake and acute kidney injury. The patient was found to have lithium-induced junctional bradycardia. Transvenous pacing was not indicated as the patient responded to fluids and atropine and had no severe hemodynamic compromise. As his serum lithium levels decreased, the bradycardia gradually improved. His echocardiogram revealed moderate left ventricular systolic dysfunction. Workup of cardiomyopathies was negative: no obstructive coronary artery disease; viral panel, and autoimmune markers were unremarkable. Thus, his cardiomyopathy was attributed to lithium toxicity. Lithium cardiotoxicity may manifest as arrhythmias and/or cardiomyopathy. Clinicians should have a high index of suspicion for lithium cardiotoxicity due to the narrow therapeutic range of lithium.

## Introduction

Lithium has been widely and effectively used for mood disorders over the past several decades. Due to its narrow therapeutic index of 0.6-1.2 mEq/L [1], close monitoring of lithium serum levels is required. Although lithium is primarily known to cause neurological and gastrointestinal side effects, cardiac side effects have also been rarely reported. We present a unique case of lithium cardiotoxicity that presented as bradyarrhythmia and cardiomyopathy attributed to lithium cardiotoxicity.

## Case presentation

A 68-year-old man with a history of paranoid schizophrenia, bipolar disorder, hypertension, hypothyroidism and hyperlipidemia presented to the emergency room with altered mental status. His sister reported that he had decreased oral intake and dysphagia over the past several weeks. On physical examination, the patient was lethargic, with dry oral mucosa and a regular pulse of 42 bpm.

**Figure 1:**
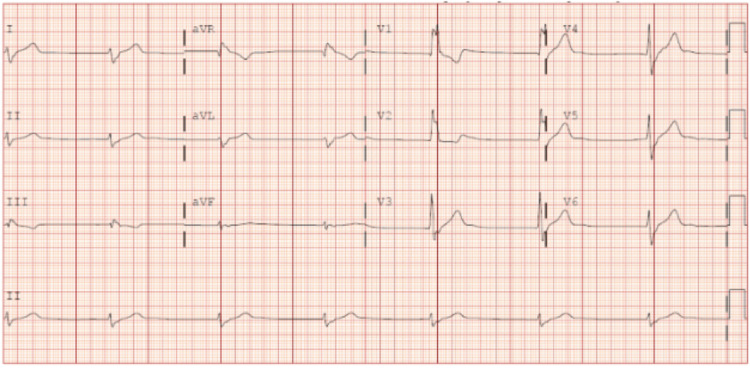
Electrocardiogram on presentation Initial electrocardiogram demonstrating a junctional escape rhythm with a right bundle morphology

Lab studies were notable for serum creatinine 1.22 mg/dL (0.6-1.1) and serum lithium 2.04 mEQ/L (0.6-1.2). Respiratory viral panel, influenza, human immunodeficiency virus, Lyme titer, and coronavirus disease 2019 (COVID-19) polymerase chain reaction (PCR) were negative. Computed tomography (CT) brain, CT angiography neck and chest X-ray revealed no acute abnormality. Electrocardiogram (ECG) revealed a junctional escape rhythm with a right bundle morphology, heart rate of 40 bpm (Figure 1).

**Figure 2:**
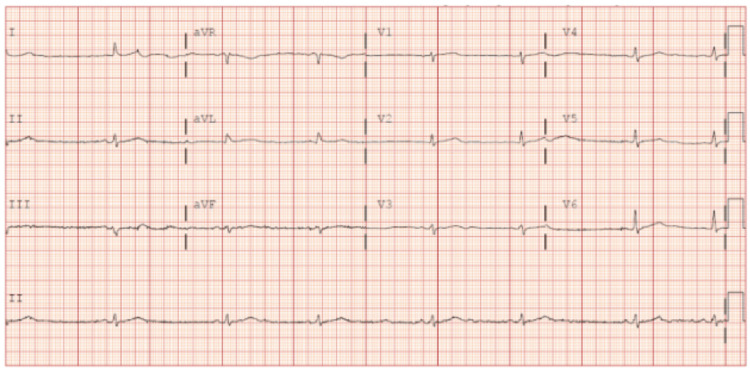
Subsequent electrocardiogram Subsequent electrocardiogram revealing sinus bradycardia

The patient was treated with intravenous fluids and atropine injection. Serial ECGs revealed sinus bradycardia with no sinus pauses (Figure 2).

Home medications included aspirin, enalapril, atorvastatin, haloperidol, lithium, trihexyphenidyl, valbenazine, and levothyroxine. Lithium toxicity was strongly suspected in the setting of raised serum lithium levels, decreased oral intake and acute kidney injury. However, other causes that had to be ruled out as a possible differential included Lyme cardiotoxicity, electrolyte abnormalities (i.e hyperkalemia), hypothermia, hypothyroidism (given the patient’s past medical history, poisonings and toxic exposures [AV nodal blocking agents, digoxin]). Echocardiogram revealed moderately reduced left ventricular systolic function (ejection fraction 40%) with no regional wall motion abnormalities. Ischemic workup with CT coronary angiogram revealed only mild atherosclerotic disease and did not account for the cardiomyopathy.

Lithium was discontinued. The patient was treated with supportive therapy and telemetry monitoring. The patient did not meet criteria for hemodialysis or transvenous pacing. A detailed workup to rule out other causes of sinus node dysfunction and cardiomyopathy was completed as above, which was largely unremarkable. Supportive therapy with adequate hydration to maintain hemodynamics, resulted in decreasing lithium levels and eventual resolution of symptoms and improvement of bradycardia. The patient’s heart rate improved to 50-65 bpm returning to sinus rhythm as serum lithium levels continued to decrease. He was treated with an angiotensin receptor blocker for the cardiomyopathy. He was discharged following an uneventful hospital course and a psychiatric follow-up to replace lithium with an alternative therapy.

## Discussion

Lithium is the first-line drug for long-term treatment of bipolar disorder [2]. However, its narrow therapeutic index of 0.6-1.2 mEq/L mandates close monitoring of its serum levels. Apart from its other systemic side effects including gastrointestinal, neurological, renal, thyroid, cognitive and dermatological effects, lithium has been reported to cause various electrocardiographic (ECG) changes. These can range from T wave inversions to arrhythmias and complete heart block. A systematic review in 2017 that included data from 26 case reports, eight clinical studies and 12 review articles demonstrated various electrocardiographic manifestations of lithium toxicity. These can include sinus bradycardia, PR prolongation, QT prolongation, sinoatrial blocks, incomplete bundle branch block, the Brugada pattern, and ventricular tachyarrhythmias [3]. Although the exact mechanism of this effect is not entirely understood, it is thought that lithium exerts its effects on the voltage-gated sodium channels, which alters the physiological stability of the cardiomyocyte cell membrane. This interferes with the electrical impulse propagation and depolarization causing the changes seen on the electrocardiogram [4].

Previously reported cases of bradyarrhythmia secondary to lithium toxicity have discussed various treatment options based on severity. Management of mild cases includes discontinuation of lithium and supportive treatment [5]. Moderate cases require intravenous fluids, bowel irrigation or gastric lavage [6]. Severe cases may require urgent dialysis and subsequent temporary or permanent pacemaker if refractory [7]. Our patient was treated with supportive therapy as he did not meet criteria for hemodialysis or a pacemaker. The indications of hemodialysis in lithium toxicity include hemodynamic instability, serum lithium levels >4 mEQ/L, severe neurologic dysfunction or renal insufficiency not responding to intravenous hydration [8]. Similarly, the indications for transvenous pacing include hemodynamic compromise, evidence of high-grade atrioventricular, infra-Hissian or complete heart block and absence of reversible causes [9].

In addition to the aforementioned cardiac side effects, some cases of edema due to new-onset heart failure have also been observed in patients using lithium carbonate [10]. In most of these cases, lithium levels were within the therapeutic range at the upper limits, and the cardiotoxicity resolved after discontinuing lithium treatment. The precise mechanisms underlying these symptoms are not fully understood, but potential hypotheses include myofibrillar degeneration with infiltration of lymphocytes into the heart muscle, interference with the influx of calcium ions in pacing cells, and adrenergic stimulation [11,12].

Our patient’s history of lithium use was of five years duration, with a dosage of 600 mg daily for the past six months. His echocardiogram revealed moderately reduced left ventricular systolic function with no regional wall motion abnormalities. Ischemic workup with CT coronary angiogram revealed only mild atherosclerotic disease. Detailed workup including viral panel and autoimmune markers was unremarkable. Therefore, in the absence of any other probable cause of cardiomyopathy, this patient’s cardiomyopathy is likely related to chronic lithium use. Extensive review of literature showed only a handful of published case reports on lithium-induced cardiomyopathy. One patient developed acute left ventricular dysfunction as a result of lithium intoxication which presented as arrhythmia [12]. Another patient presented with dilated cardiomyopathy after long-term lithium therapy for 16 years [13]. The mechanism may be attributed to the increased urinary and serum norepinephrine levels, which are seen with chronic lithium use [14]. The increase in catecholamines is supported by evidence from animal studies revealing dose-dependent increases in plasma catecholamine levels from the adrenal gland of rats, perfused adrenal gland of cats, and cultured bovine adrenal medullary cells in response to lithium [15]. This mechanism is also implicated in Takotsubo cardiomyopathy, which has been seen in association with lithium intoxication secondary to increased peripheral catecholamines [15]. Myofibrillar degeneration with myocardial lymphocyte cell infiltrates and fibrosis has also been proposed as a potential pathophysiological mechanism leading to lithium-induced cardiomyopathy [13,14].

## Conclusions

Clinicians should have a high index of suspicion for lithium cardiotoxicity due to the narrow therapeutic range of lithium. Lithium cardiotoxicity may manifest as arrhythmias and/or cardiomyopathy. While there is limited data confirming the link between lithium toxicity and development of cardiomyopathy, it is still crucial to consider the potential risk in patients receiving long-term lithium therapy. It is essential to obtain a detailed patient history to identify patients at risk, monitor for side effects while on therapy, and to refer to a specialist in a timely manner to prevent and treat complications.
